# Emergence and establishment of KPC-2-producing ST11 *Klebsiella pneumoniae* in a general hospital in Shanghai, China

**DOI:** 10.1007/s10096-017-3131-4

**Published:** 2017-12-27

**Authors:** Jingxian Liu, Jing Yu, Feng Chen, Jiajia Yu, Patricia Simner, Pranita Tamma, Ying Liu, Lisong Shen

**Affiliations:** 1 Department of Clinical Laboratory, Xin Hua Hospital, Shanghai Jiao Tong University School of Medicine, Shanghai, China; 2 Department of Pathology, Johns Hopkins Hospital, Johns Hopkins University School of Medicine, Baltimore, MD USA

## Abstract

The aim of this study was to investigate the characteristics of carbapenem-resistant *Klebsiella pneumoniae* (CRKP) collected during an outbreak in a Chinese teaching hospital and to provide insights into the prevention and control of nosocomial infection. We collected unique CRKP clinical isolates from 2009 to 2013. Antibiotic-resistant genes were identified by polymerase chain reaction (PCR) and sequencing. The isolates were typed using pulsed-field gel electrophoresis (PFGE) and multilocus sequence typing (MLST). Plasmids were classified using a PCR-based incompatibility/replicon typing method and a replicon sequence typing method. Conjugation experiments were performed to evaluate the transferability of carbapenem-resistant genes. Whole genome sequencing (WGS) was conducted to further investigate the genetic background of the isolates. Infection control practices were reviewed throughout the study period. *Klebsiella pneumoniae* sequence type (ST) 11 emerged in 2010 and acquired the *bla*_KPC-2_ gene by 2011. From 2011 to 2013, ST11 KPC-2-producing CRKP (G type) prevailed as the most common CRKP in our hospital, causing a prolonged outbreak. The majority of these CRKP strains possess an IncFII plasmid, with Tn1721-*bla*_KPC-2_-ΔTn3-IS26 bearing the genetic structure for *bla*_KPC-2_. Infection prevention control measures available at the time contained the initial outbreak, but had no effect on the spread of CRKP later. This study demonstrated the seriousness concerning the spread of KPC-2-producing ST11 CRKP in a Chinese hospital, indicating that current prevention and control strategies for carbapenem-resistant Enterobacteriaceae (CRE) nosocomial infection need to be investigated and adjusted.

**Electronic supplementary material:**

The online version of this article (10.1007/s10096-017-3131-4) contains supplementary material, which is available to authorized users.

## Introduction

The emergence and transmission of carbapenem-resistant Enterobacteriaceae (CRE) over the past two decades has attracted worldwide attention, for its indication that most currently available broad-spectrum antibiotics may no longer be a therapeutic option for some patients [1]. CRE have disseminated rapidly around the world, resulting in high morbidity and mortality [2]. To combat this problem, many healthcare facilities have implemented aggressive infectious control practices when patients with CRE are identified [3, 4]. However, as intensive infection control measures (ICMs) can require significant resources and personnel efforts, not all hospitals can implement potential solutions, such as individual patient rooms and dedicated nurses for CRE-positive patients, particularly when healthcare facilities are insufficient in developing countries. In this study, we describe the emergence and establishment of carbapenem-resistant *Klebsiella pneumoniae* (CRKP) sequence type (ST) 11 within our hospital and the measures taken to control the outbreak.

## Materials and methods

### Setting and isolate collection

Xinhua Hospital is a 2000-bed teaching hospital located in Shanghai, China. All nonduplicate *K. pneumoniae* clinical isolates meeting the Clinical and Laboratory Standards Institute (CLSI) criteria for CRE from June 2009 to December 2013 were stored in glycerol at − 80 °C for subsequent testing. From 2014 to 2016, CRKP clinical isolates were continuously collected and stored for outbreak analysis. The KPC-2 gene was screened in theses isolates, and multilocus sequence typing (MLST) analysis was performed for KPC-2-positive strains. Genus and species were identified using the VITEK 2 Compact system (bioMérieux, Marcy l’Étoile, France), and organism identification was verified using Microflex™ matrix-assisted laser desorption/ionization time-of-flight mass spectrometry (MALDI-TOF MS; Bruker Daltonics, Bremen, Germany). Antibiotic susceptibility tests for tigecycline and colistin were performed using a broth microdilution method, and interpreted by Food and Drug Administration (FDA) and European Committee on Antimicrobial Susceptibility Testing (EUCAST) criteria, respectively, while the remaining antibiotics in Table S2 were tested using the VITEK 2 Compact system combined with a disk diffusion method and interpreted by CLSI criteria.

### PCR amplification and sequencing

Frozen isolates were thawed and subcultured prior to DNA extraction using the Rapid Bacterial Genomic DNA Isolation Kit (Sangon Biotech, Shanghai, China). To identify the presence of plasmid-mediated AmpC β-lactamase genes (*bla*_CIT_, *bla*_EBC_, *bla*_ACC_, *bla*_DHA_, *bla*_FOX_, and *bla*_MOX_), extended-spectrum β-lactamase (ESBL) genes (*bla*_CTX-M_, *bla*_TEM_, and *bla*_SHV_), and carbapenemase genes (*bla*_KPC_, *bla*_GES_, *bla*_IMP_, *bla*_VIM_, *bla*_NDM_, and *bla*_OXA_), DNA was amplified by polymerase chain reaction (PCR) as previously described (Table S1). Positive amplification products were sent to Sangon Biotech for sequencing.

### PFGE analysis

Pulsed-field gel electrophoresis (PFGE) typing of clinical CRKP isolates was performed as previously described [5]. The PFGE patterns were analyzed by BioNumerics software (Applied Maths NV, Sint-Martens-Latem, Belgium) using the Dice similarity coefficient. Strains were considered to be of the same clone if they possessed ≥ 95% genetic similarity in the PFGE profiles. Clusters were defined as DNA patterns sharing ≥ 75% similarity [5].

### MLST analysis

Gene amplification and sequencing of seven housekeeping genes of *K. pneumoniae* (*gapA*, *infB*, *rpoB*, *phoE*, *mdh*, *pgi*, and *tonB*) were performed following a protocol provided online (http://bigsdb.pasteur.fr/klebsiella). Allele and STs were determined according to the MLST database of the Institut Pasteur.

### Plasmid analysis

Plasmids were classified using the PCR-based incompatibility/replicon typing method previously described [6]. A replicon sequence typing (RST) method was applied to discriminate IncF plasmid variants [7].

### Conjugation for *bla*_KPC-2_ transferability

Conjugation experiments were performed using KPC-2-producing strains as donors and *Escherichia coli* J53 as the recipient [8]. Overnight cultures of donor and recipient were mixed at a 1:1 ratio, and the broth was incubated at 35 °C for 12 h. Following incubation, 100 μL of the conjugation mixture was inoculated in Mueller–Hinton medium containing 2 μg/mL imipenem and 125 μg/mL sodium azide, and incubated at 35 °C overnight. The minimum inhibitory concentration (MIC) for imipenem was determined and PCR for *bla*_KPC-2_ was performed on the transconjugant.

### WGS

Seven isolates were randomly selected from KPC-2-producing ST11 CRKP, along with one isolate from non-KPC-2-producing ST11 CRKP strains. Total DNA was extracted using the SDS–proteinase K method. Whole genome sequencing (WGS) was performed with the Illumina HiSeq 2500 system (Illumina, USA). SPAdes (version 3.9.0) was used to assemble the sequences. The plasmid sequences were assembled using plasmidSPAdes (version 3.9.0). Protein function annotation was achieved through BLAST (version 2.2.31+) and HMMER (version 3.1b1). MASH version 1.0.1 was used to compare genes of tested isolates with assembled genomes in the NCBI database. The mash distance was calculated to draw the phylogeny tree using unweighted pair group method with arithmetic mean (UPGMA).

### Patient information

Clinical information including age, location in hospital, previous carbapenem use, and isolation status during hospitalization was collected on all patients who contributed isolates.

## Results

### Patient characteristics

Eighty-five strains of CRKP were isolated from clinical samples during the period from 2009 to 2013 and were subjected to further investigation. These 85 strains were isolated from various clinical sources from 75 unique patients. The most common sources were sputum (48%), urine (20%), and blood (13%). The majority of CRKP were isolated from the surgical intensive care unit (SICU) (0.6 per bed), the emergency department (0.28 per bed), and the geriatric department (0.18 per bed). Most of the patients were either very young or very old, with 21% under 1 year of age and 60% over 70 years of age. Forty-nine (65%) patients accepted traumatic mechanical ventilation before CRKP isolation. Fifty-six patients (75%) were treated with a carbapenem in the month prior to CRKP isolation.

### Isolate characteristics

The antimicrobial susceptibility testing profiles are summarized in Table S2. Seventy-four (87%) of the 85 isolates were resistant to all carbapenems tested (ertapenem, meropenem, and imipenem), but were all still sensitive to tigecycline and colistin. Next, 54.1% of the 85 isolates can be defined as extensively drug-resistant (XDR) strains, and the remaining 45.9% can be defined as multidrug-resistant (MDR) strains [9].

**Fig. 1:**
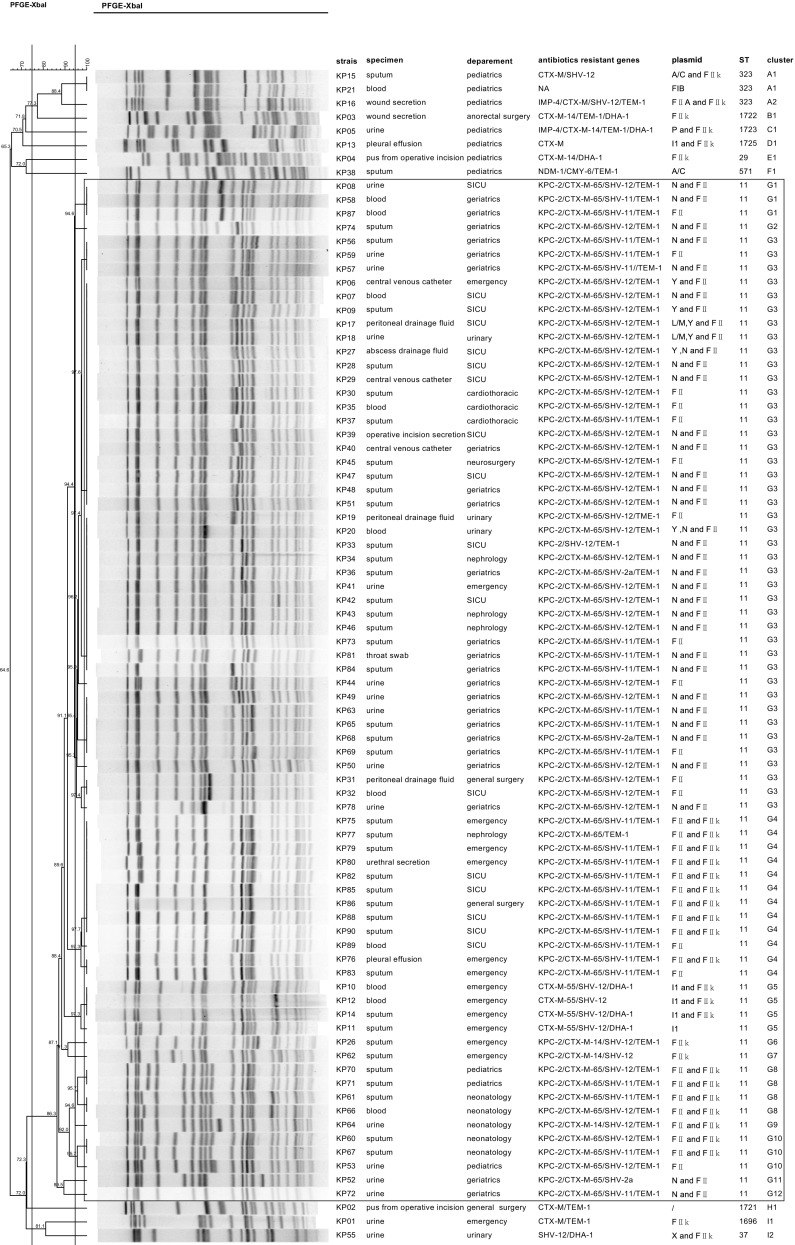
Pulsed-field gel electrophoresis (PFGE) dendrogram of carbapenem-resistant *Klebsiella pneumoniae* (CRKP) isolates from 2009 to 2013. All the isolates enclosed by the rectangle belong to the G cluster as well as sequence type (ST) 11

Seventy-three (86%) strains were carrying a carbapenemase gene. Three types of carbapenemases were detected, including KPC-2, IMP-4, and NDM-1. KPC-2 was the most common type, comprising 95% of carbapenemases identified. Additionally, almost all strains (98%) carried one or more ESBL genes, including 95% *bla*_CTX-M_, 89% *bla*_SHV_, and 87% carrying a narrow-spectrum β-lactamase *bla*_TEM-1_. Plasmid-mediated AmpC genes were present in 9.4% of isolates, while seven strains harbored *bla*_DHA-1_ and one strain harbored *bla*_CMY-6_ (Fig. 1).

A total of ten STs were identified within 85 strains by MLST, and 87% (74/85) of the strains belonged to ST11. Other STs, such as ST571, ST29, ST37, ST323, ST1696, ST1721, ST1722, ST1723, and ST1725, were also detected. The last five types listed were new STs discovered in this study and have been submitted to the Institut Pasteur for registration. The 85 strains were divided into nine clusters by PFGE, ranging from clusters A to I (Fig. 1). MLST and PFGE yielded similar results, with all G type strains belonging to ST11. G cluster strains can be further divided into 12 types, with G3 being the main clone disseminated throughout the eight departments of the hospital. Approximately 95% (70/74) of ST11 strains harbored the *bla*_KPC-2_ gene, along with one or more ESBL genes.

Replicon typing demonstrated that the CRKP isolates harbored one to three plasmids. Ninety-seven percent of KPC-2 producing ST11 strains harbored IncFII plasmid, and 76% (53/70) coharbored an additional plasmid type, generally IncN or IncFIIk. All the non-KPC-2-producing ST11 isolates had an IncI1 plasmid, while plasmid types were variable in non-ST11 CRKP strains. All strains from the neonatology department and most of the strains isolated from the pediatrics (70%) and emergency (71%) wards had an IncFIIk plasmid, while most isolates from the geriatrics department (74%) coharbored IncN and IncFII plasmid (Fig. 1). Only 3 out of 70 KPC-2-producing strains successfully transmitted *bla*_KPC-2_ to *E. coli* J53. However, the IncFII replicon was not detected in those transconjugants.

**Fig. 2:**
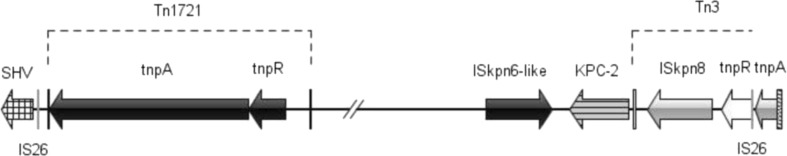
Genetic environment of the *bla*_KPC-2_ gene in this study. Genes are depicted as arrows according to the direction of transcription. Inverted repeats are indicated by color: Tn1721 (black), Tn3 (white), and IS26 (gray). *bla*_KPC_ is gray with horizontal lines

The characteristics of eight ST11 CRKP isolates using WGS are presented in Table S3. All eight strains harbored several antibiotic-resistant genes, including ARR-3, aac(6′)Ib-cr, aadA16, aadA2, catA2, dfrA27, fosA, oqxA, oqxB, rmtB, sul1, tet(A), and β-lactamase genes. Non-KPC-2-producing ST11 and KPC-2-producing ST11 isolates were not closely related (Fig. S1). The *bla*_KPC-2_-bearing genetic structure of these seven KPC-2-producing ST11 CRKP was Tn1721-*bla*_KPC-2_-ΔTn3-IS26 (Fig. 2).

### Outbreak characteristics

The first CRKP isolate was detected in June 2009. From June 2009 to September 2010, all CRKP isolates collected were non-ST11. ST11 strains were first discovered in September 2010, which quickly became the main epidemic clone in the hospital. During the early stage of the epidemic, some ST11 isolates were non-KPC-producing strains; however, after February 2011, all ST11 CRKP isolates from clinical samples were KPC-producing. Also, 51% (38/75) of patients infected with CRKP died during their hospitalizations. Surveillance measures for the early screening of high-risk patients or patients transferred from facilities known to have CRE did not occur upon admission during the study period, nor were patients placed on contact isolation when CRKP were identified. The epidemic was contained in 2012 after education about CRE, strict adherence to proper hand hygiene, and compliance with contact precautions. However, in 2013, the situation worsened again.

**Fig. 3:**
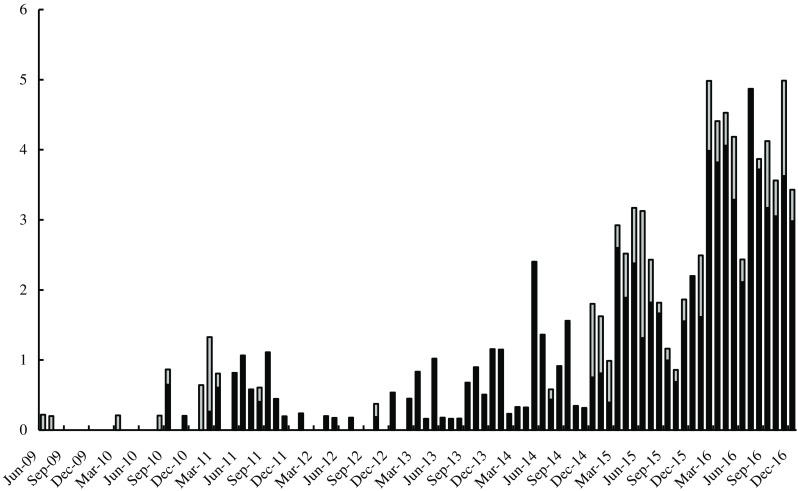
Distribution of CRKP by sequence type (ST) from 2009 to 2016. The dark color represents ST11 KPC-2-producing strains and the gray color represents non-ST11 strains, also non-KPC-producing. The *x*-axis is months and the *y*-axis is no. of events/10^4^ patient-days

Five hundred and thirty-five CRKP were isolated from 428 patients from January 2014 to December 2016, with the CRKP levels growing rapidly every year (Fig. 3). The most common sample sources were sputum (42%), urine (14%), trachea cannula (13%), and blood (9%). Fifty-five percent (235/428) of patients accepted traumatic mechanical ventilation before CRKP isolation, which was not significantly different from the 2009 to 2013 data (χ^2^ = 2.82, *p* = 0.09).

The Wilcoxon signed-rank test was performed to detect the efficiency of ICMs. Nosocomial infection control was defined as intervention and the period during the initial outbreak of CRKP to the time before intervention (September 2010 to November 2011) was defined as a control group. The isolation rate (number of CRKP isolated per month per patient-day) after intervention was compared to the control group for each year. A statistically significant decrease in the isolation rate was observed for the first two years (*p* = 0.0307), indicating that the ICMs may have helped the situation. Disappointingly, the ICMs gradually became less effective in the following years.

## Discussion

We report an outbreak of CRKP ST11 in a Chinese hospital over a 4-year period of time. At the beginning of the outbreak, ST11 *K. pneumoniae* isolates were generally non-KPC-producing and susceptible to several antibiotics. However, in recent years, these isolates became increasingly resistant to multiple drugs and began harboring KPC-2 carbapenemase. Phylogenetic analysis of the ST11 CRKP revealed that non-KPC-2-producing CRKP was distant from the KPC-2 producing strains.

ST11 CRKP was disseminated widely across China and associated with *bla*_KPC-2_ dissemination [10]. The genetic characteristics of KPC-2-producing strains in this study are similar to the isolates from other areas of China [11, 12]. The transmission of *bla*_KPC-2_ can be mediated by different molecular mechanisms, but the factors contributing to the epidemiological success of KPC-producing *K. pneumoniae* clonal complex (CC) 258 is unclear [13]. Recently, Liang et al. [14] found that the KPC-2-producing ST11 *K. pneumoniae* can code an anti-restriction protein KlcA_HS_, which may help prevent the host bacteria from recognizing and incising foreign DNA by disrupting the restriction modification (RM) systems. The strains in this study also have *bla*_KPC-2_ and KlcA_HS_ simultaneously (data not shown). Besides, the strains investigated in this study possess several plasmid-orientated antibiotic genes and revealed low conjugation efficiency, indicating their stability. Furthermore, our data showed that the first emergence of ST11 CRKP without KPC-2 was around January to February 2011, and was never detected during the outbreak of KPC-2-producing ST11 strains. We hypothesize that the ST11 *K. pneumoniae* possess a powerful ability to acquire antibiotic-resistant genes actively and conserve them. This characteristic may have contributed to their survival in the antibiotics-inundated hospital environment.

Upon further investigation, it was discovered that certain plasmid-bearing KPC-2-producing CRKP were predominant in particular wards. The geriatrics ward mostly had *K. pneumoniae* isolates with IncFII and IncN plasmids, while all of the strains isolated from the neonatology wards had both IncFII and IncFIIk replicons. This finding suggests that two successful KPC-2-producing ST11 clones were established in our hospital setting. There may be a particular gene, or genes, on the IncN plasmid that was responsible for those strains disseminating through the geriatric ward compared to those strains found in the neonatology wards.

In general, the epidemiological trend of CRKP in our hospital can be divided into three stages. From June 2009 to August 2010, only four non-ST11 CRKP were isolated. Beginning in September 2010, ST11 strains began to be isolated, but the genetic background of the isolates remained diverse until March 2011. By April 2011, almost all isolates were ST11. Based on this finding, we hypothesize that ST11 was a successful clone that established itself in our hospital setting in 2010. In 2011, it appears that the ST11 clone either acquired the KPC-2-bearing plasmid or a KPC-2-producing ST11 strain was introduced to our hospital and successfully disseminated and established itself.

Until 2011, there were minimal ICMs implemented to stop the spread of CRKP in our hospital. However, at the end of 2011, the infection control department began education related to preventing the spread of CRE, became more aggressive about hand hygiene compliance, and began routine contact precautions for patients with CRE isolates. Supervisory staff was appointed to oversee hand hygiene and contact precaution compliance. The isolated rate of CRKP decreased drastically afterwards. However, the isolation rate of CRKP began increasing every year beginning in 2013. Noticeably, CRKP isolated from trachea cannula experienced the greatest increase in the last 3 years (2014 to 2016). This finding means that the proportion of unplanned extubation caused by CRKP infection increased significantly, indicating that CRKP may become a considerably more difficult problem in the future.

Our data show that the ICMs may have had a short-term effect from 2012 to 2013, but gradually lost efficacy in the following years. We believe that the KPC-2-producing ST11 clone may possess features which make it easy to colonize and hard to be eradicated. These features may facilitate the bacterial load reaching a certain level and then triggering an unmanageable outbreak. Additionally, standardized systems for the management of CRKP carriers or infected populations are still deficient in China at this time. Considering the severe situation of CRKP prevalence throughout China and the mobility of CRKP carriers or infected populations, the epidemic-causing clone may be introduced to our hospital and contribute to the rise of CRKP.

According to our results and the current situation in China, we propose that nosocomial infection control strategies for CRE should be reevaluated and adjusted. Munoz-Price and Quinn [3] summarized several infection control bundles, almost all of which include strict hand hygiene and implementation of contact precautions. However, several reports suggest that CRE would not be effectively controlled by these measures alone [15, 16]. Early identification of symptomatic or asymptomatic carriers to identify a silent epidemic and the subsequent cohorting of patients may be important for CRE control [17]. Recently, more evidence has emerged demonstrating that multifaceted hospital-wide intervention programs are required to control CRE in the hospital setting. These programs include improved hand hygiene compliance, cohort programs with dedicated nursing staff, addition of regular screening in high-risk departments, and carbapenem restriction [16, 18, 19]. Gohil et al. [20] suggested that proactive strategies, such as chlorhexidine bathing, should be considered in the setting of a regional collaborative. Educational activities along with reasonable antimicrobial stewardship has also been shown to be effective in reducing the incidence of CRKP bloodstream infections [21]. However, CRKP reduced susceptibility to commonly used biocides was also reported in recent years, which may pose a new challenge to infection control [22].

Several lessons were learned from our outbreak. First, we had failed to educate our medical staff on the importance of CRE and both its associated morbidity and mortality, as well as the rapidity with which they can disseminate within institutions. Second, at the onset of the outbreak, strict contact isolation and cohorting of patients should have been implemented. Third, hand washing supervisors should have been hired early in the outbreak to enforce appropriate and routine hand hygiene. Fourth, the notable use of broad-spectrum antibiotics in patients involved in the outbreak underscored the importance of antibiotic stewardship programs for overseeing appropriate antibiotic use. Finally, early consideration of active surveillance may have identified the magnitude of the epidemic early on, with the simultaneous implementation of all aforementioned measures having helped to halt this outbreak shortly after it was recognized.

The prevalence of CRKP, especially the KPC-2-producing ST11 clone, has become extremely serious in China in recent years. It is important to identify the intrinsic factors which caused the rapid dissemination of ST11 and to focus on CRKP infectious control as well as surveillance.

## Electronic supplementary material

Below is the link to the electronic supplementary material.

ESM 1 (DOC 772 kb)
