# Supplementary material for: Emergence and establishment of KPC-2-producing ST11 Klebsiella pneumoniae in a general hospital in Shanghai, China
Source: Eur J Clin Microbiol Infect Dis. 2017 Dec 27;37(2):293–9. doi: 10.1007/s10096-017-3131-4 (PMC5780533; doi:10.1007/s10096-017-3131-4)

**Table S1** Primers Sequences for PCR Amplification

|  | genes | Primers sequence | Product size | references |
| --- | --- | --- | --- | --- |
| Carbapenemases | KPC | F：GCTACACCTAGCTCCACCTTC  R：ACAGTGGTTGGTAATCCATGC | 989bp | [1] |
|  | GES | F：AGCGACAATGGGGCTACTAAC  R：GTGTAATAACTTGACCGACAGAGG | 465bp | This study |
|  | IMP | F：CTACCGCAGCAGAGTCTTTG  R：AACCAGTTTTGCCTTACCAT | 587bp | [2] |
|  | VIM | F：AGTGGTGAGTATCCGACAG  R：ATGAAAGTGCGTGGAGAC | 261bp | [3] |
|  | NDM-1 | F：GAGCACCGCATTAGCCGCTG  R： GCTATCGGGGGCGGAATGG | 727bp | This study |
|  | OXA-48 | F：TTGGTGGCATCGATTATCGG  R： GAGCACTTCTTTTGTGATGGC | 744bp | [4] |
| AmpC lactamases | CIT | F:TGGCCAGAACTGACAGGCAAA R:TTTCTCCTGAACGTGGCTGGC | 462bp | [5] |
|  | DHA | F:AACTTTCACAGGTGTGCTGGGT R:CCGTACGCATACTGGCTTTGC | 405bp |  |
|  | EBC | F:TCGGTAAAGCCGATGTTGCGG R:CTTCCACTGCGGCTGCCAGTT | 302bp |  |
|  | MOX | F:GCTGCTCAAGGAGCACAGGAT R:CACATTGACATAGGTGTGGTGC | 520bp |  |
|  | FOX | F:AACATGGGGTATCAGGGAGATG R:CAAAGCGCGTAACCGGATTGG | 190bp |  |
|  | ACC | F:AACAGCCTCAGCAGCCGGTTA R:TTCGCCGCAATCATCCCTAGC | 346bp |  |
| ESBLs | CTX-M | F: GCCTCGTGAAGAAGGTGTTG R: CGTATTGGGAGTTTGAGATGG | 1110bp | This study |
|  | TEM | F: CCCCTATTTGTTTATTTTTC R: GACAGTTACCAATGCTTAATCA | 941bp | [6] |
|  | SHV | F: GCCGGGTTATTCTTATTTGTCGC R: TCTTTCCGATGCCGCCGCCAGTCA | 1012bp | [6] |

**References**

1. Smith Moland E HN, Herrera VL, Black JA, Lockhart TJ, Hossain A, Johnson JA, Goering RV, Thomson KS (2003) Plasmid-mediated, carbapenem-hydrolysing beta-lactamase, KPC-2, in Klebsiella pneumoniae isolates. J Antimicrob Chemother 51 (3):711-714.

2. Senda K AY, Ichiyama S, Nakashima K, Ito H, Ohsuka S, Shimokata K, Kato N, Ohta M. (1996) PCR detection of metallo-beta-lactamase gene (blaIMP) in gram-negative rods resistant to broad-spectrum beta-lactams. J Clin Microbiol 34 (12):2909-2913.

3. Tsakris A PS, Woodford N, Palepou MF, Babini GS, Douboyas J, Livermore DM. (2000) Outbreak of infections caused by Pseudomonas aeruginosa producing VIM-1 carbapenemase in Greece. J Clin Microbiol 38 (3):1290-1292

4. Poirel L HC, Tolün V, Nordmann P. (2004) Emergence of oxacillinase-mediated resistance to imipenem in Klebsiella pneumoniae. Antimicrobial agents and chemotherapy 48 (1):15-22.

5. Pérez-Pérez FJ HN (2002) Detection of plasmid-mediated AmpC beta-lactamase genes in clinical isolates by using multiplex PCR. J Clin Microbiol 40 (6):2153-2162.

6. Yan JJ WS, Tsai SH, Wu JJ, Su IJ. (2000) Prevalence of SHV-12 among clinical isolates of Klebsiella pneumoniae producing extended-spectrum beta-lactamases and identification of a novel AmpC enzyme (CMY-8) in Southern Taiwan. Antimicrob Agents Chemother 44 (6):1438-1442.

**Table S2** Antimicrobial susceptibility testing results of carbapenem-resistant *Klebsiella pneumoniae* isolates from 2009 to 2013.

| Antibiotics | S | I | R |
| --- | --- | --- | --- |
| Imipenem ^a^ | 8 (9.4%) | 3(3.5%) | 74(87.1%) |
| Meropenem ^b^ | 8(9.4%) | 0(0.0%) | 77(90.6%) |
| Ertapenem ^a^ | 0(0.0%) | 0(0.0%) | 85(100%) |
| Ampicillin ^a^ | 0(0.0%) | 0(0.0%) | 85(100%) |
| Piperacillin ^b^ | 0(0.0%) | 0(0.0%) | 85(100%) |
| Cefaclor ^b^ | 0(0.0%) | 0(0.0%) | 85(100%) |
| Cefmetazole ^b^ | 0(0.0%) | 0(0.0%) | 85(100%) |
| Cefuroxime ^b^ | 0(0.0%) | 0(0.0%) | 85(100%) |
| Cefazolin ^a^ | 0(0.0%) | 0(0.0%) | 85(100%) |
| Cefotetan ^a^ | 0(0.0%) | 1(1.2%) | 84(98.8%) |
| Ceftriaxone ^a^ | 0(0.0%) | 0(0.0%) | 85(100%) |
| Ceftazidime ^a^ | 0(0.0%) | 0(0.0%) | 85(100%) |
| Cefotaxime ^b^ | 0(0.0%) | 0(0.0%) | 85(100%) |
| Cefepime ^a^ | 0(0.0%) | 0(0.0%) | 85(100%) |
| Aztreonam ^a^ | 0(0.0%) | 0(0.0%) | 85(100%) |
| Ampicillin/Sulbactam ^a^ | 0(0.0%) | 0(0.0%) | 85(100%) |
| Piperacillin/Tazobactam ^a^ | 4(4.7%) | 1(1.2%) | 80(94.1%) |
| Cefoperazone/Sulbactam ^b^ | 3(3.5%) | 2(2.4%) | 80(94.1%) |
| Tobramycin ^a^ | 6(7.1%) | 6(7.1%) | 73(85.8%) |
| Gentamicin ^a^ | 7(8.2%) | 0(0.0%) | 78(91.8%) |
| Levofloxacin ^a^ | 8(9.4%) | 0(0.0%) | 77(90.6%) |
| Amikacin ^a^ | 14(16.5%) | 1(1.2%) | 70(82.3%) |
| Ciprofloxacin ^a^ | 8(9.4%) | 1(1.2%) | 76(89.4%) |
| Trimethoprim/Sulfamethoxaz ^a^ ole | 19(22.4%) | 0(0.0%) | 66(77.6%) |
| Tetracycline ^b^ | 34(40%) | 3(3.5%) | 48(56.5%) |
| Minocycline ^b^ | 23(27.1%) | 17(20%) | 45(52.9%) |
| Tigecycline ^c^ | 85(100.0%) | 0(0.0%) | 0(0.0%) |
| Fosfomycin ^b^ | 29(34.1%) | 2(2.4%) | 54(63.5%) |
| Chloramphenicol ^b^ | 9(10.6%) | 2(2.4%) | 74(87.0%) |
| Colistin ^d^ | 85(100.0%) | 0(0.0%) | 0(0.0%) |

^a^: The antimicrobial susceptibility test of these antibiotics was conducted by Vitek-2 Compact System and interpreted by CLSI criteria.

^b^: The antimicrobial susceptibility test of these antibiotics was conducted by disc diffusion method and interpreted by CLSI criteria.

^c^: For tigecycline, the antimicrobial susceptibility test was performed using broth microdilution method and interpreted by FDA criteria.

^d^: For colistin, the antimicrobial susceptibility test was performed using broth microdilution method and interpreted by EUCAST criteria.

**Table S3**. Characteristics of eight ST11 carbapenem-resistant Klebsiella pneumonia isolates using whole genome sequencing

| strains | Isolate date | patients | KPC-2 producing | contigs | Genome bases(bp) | Drug resistant genes |
| --- | --- | --- | --- | --- | --- | --- |
| kp10 | 2011/1/12 | adult | N | 109 | 5733940 | blaCTX-M-55, blaDHA-1, blaSHV-12, QnrB4, QnrS1, fosA, mph(A), oqxA, oqxB, sul1 |
| kp6 | 2010/9/19 | adult | Y | 112 | 5569296 | aadA1,blaCTX-M-65,blaKPC-2,blaSHV-12,blaTEM-1B,catA2,fosA,oqxA,oqxB,rmtB |
| kp41 | 2011/9/7 | adult | Y | 131 | 5647205 | ARR-3, aac(6')Ib-cr, aadA16, aadA2, blaCTX-M-65, blaKPC-2, blaSHV-12, blaTEM-1B, catA2, dfrA27, fosA, oqxA, oqxB, rmtB, sul1, tet(A) |
| kp56 | 2012/11/2 | adult | Y | 114 | 5656613 | ARR-3,QnrA1,aadA2,blaCTX-M-65,blaKPC-2,blaSHV-12,blaTEM-1B,catA2,dfrA27,fosA,oqxA,oqxB,rmtB,sul1,tet(A) |
| kp81 | 2013/10/11 | adult | Y | 135 | 5669340 | ARR-3,aadA16,aadA2,blaCTX-M-65,blaKPC-2,blaSHV-12,blaTEM-1B,catA2,dfrA27,fosA,oqxA,oqxB,rmtB,sul1,tet(A) |
| kp53 | 2012/7/13 | child | Y | 137 | 5507125 | aadA1,blaCTX-M-65,blaKPC-2,blaSHV-12,blaTEM-1B,catA2,fosA,oqxA,oqxB,rmtB,sul1 |
| kp60 | 2013/2/19 | child | Y | 135 | 5675402 | aadA2,blaCTX-M-65,blaKPC-2,blaSHV-12,blaTEM-1B,catA2,dfrA12,fosA,oqxA,oqxB,rmtB,strA,strB, sul2 |
| kp70 | 2013/5/27 | children | Y | 137 | 5602608 | blaCTX-M-65,blaKPC-2,blaSHV-12,blaTEM-1B,catA2,fosA,rmtB,strA,strB,sul2 |

**Fig S1**. Phylogeny tree of 8 CRKP and assembled *Klebsiella pneumoniae* genomes


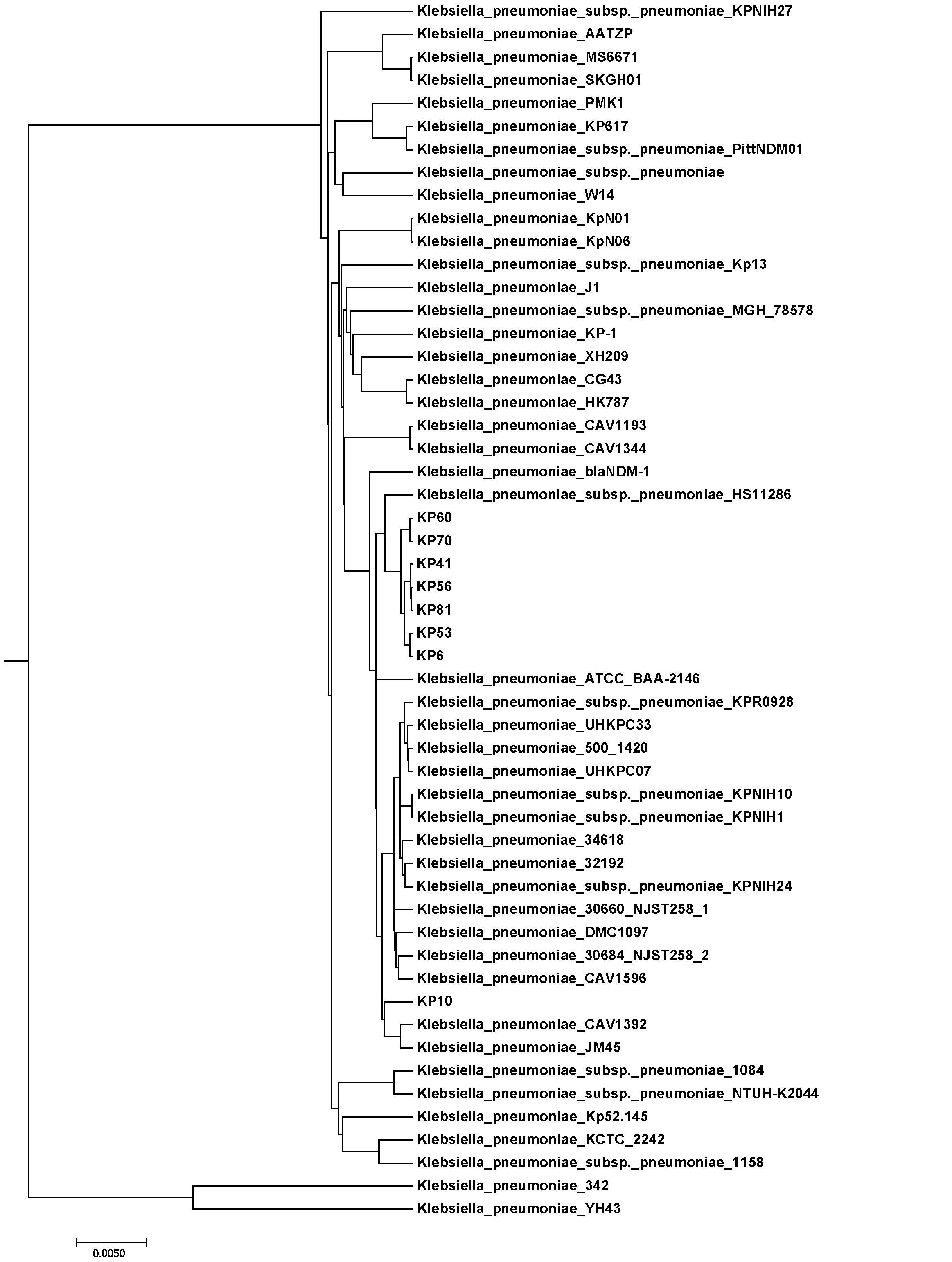

Supplement: Supplementary file 1 — (DOC 772 kb) [file 10096_2017_3131_MOESM1_ESM.doc]
